# The Fabrication of Gold Nanostructures as SERS Substrates for the Detection of Contaminants in Water

**DOI:** 10.3390/nano14181525

**Published:** 2024-09-20

**Authors:** Cristhian A. Visbal, Wilkendry Ramos Cervantes, Lorena Marín, John Betancourt, Angélica Pérez, Jesús E. Diosa, Luis Alfredo Rodríguez, Edgar Mosquera-Vargas

**Affiliations:** 1 Grupo de Películas Delgadas, Departamento de Física, Universidad del Valle, Santiago de Cali 760032, Colombia; cristhian.visbal@correounivalle.edu.co (C.A.V.); betancourt.john@correounivalle.edu.co (J.B.); 2 Institución Educativa Número Dos, Maicao 442001, Colombia; ramos.wilkendry@iedosmaicao.edu.co; 3 Centro de Excelencia en Nuevos Materiales (CENM), Universidad del Valle, Santiago de Cali 760032, Colombia; angelica.perez@correounivalle.edu.co (A.P.); jesus.diosa@correounivalle.edu.co (J.E.D.); luis.a.rodriguez@correounivalle.edu.co (L.A.R.); edgar.mosquera@correounivalle.edu.co (E.M.-V.); 4 Grupo de Óptica Cuántica, Departamento de Física, Universidad del Valle, Santiago de Cali 760032, Colombia; 5 Grupo de Transiciones de Fase y Materiales Funcionales, Departamento de Física, Universidad del Valle, Santiago de Cali 760032, Colombia

**Keywords:** gold nanostructures, surface plasmon, Rhodamine B, SERS, dewetting

## Abstract

Gold nanostructures (AuNSs) were used to fabricate surface-enhanced Raman spectroscopy (SERS) substrates. These AuNSs were produced using the solid-state dewetting method from thin films. The fragmentation process was studied at 300 °C, with durations of thermal treatment of 1, 3, 6, and 12 h. These SERS substrates were then employed to detect Rhodamine B (RhB) as the model analyte, simulating a contaminant in the water at a concentration of 5 ppm. The morphology of the AuNSs was examined using SEM, which revealed a spheroidal shape that began to coalesce at 12 h. The size of the AuNSs was estimated to range from 22 ± 7 to 24 ± 6 nm, depending on the annealing time. The localized surface plasmon resonance of the AuNSs was determined using absorption spectroscopy, showing a shift as the annealing time increased. The SERS signals of RhB adsorbed on the AuNS substrates were validated by performing a 10 × 10 point map scan over each sample surface (1, 3, 6, and 12 h), and a comparative analysis showed no significant differences in the positions of the bands; however, variations in intensity enhancement ranged from 5 to 123 times at 6 and 1 h, respectively.

## 1. Introduction

The significant rise in mining, pharmaceutical, industrial, and agricultural activities has raised concerns about the widespread introduction of emerging contaminants into aquatic ecosystems [1,2,3,4]. These emerging contaminants are defined as those produced by a wide variety of products for daily human and animal use. They are characterized by being found in very low concentrations (in the order of ng/L and µg/L) and are, therefore, unregulated, even though their potential danger to ecosystems and human health has been observed [2,3,5,6,7,8].

The increasing presence of these contaminants in water not only threatens the health of ecosystems but also the health and well-being of communities that depend on water as a vital resource. In this context, the accurate and timely detection of contaminants has become crucial to mitigate the associated risks and safeguard water quality for present and future generations.

Conventionally, sophisticated methods such as high-performance liquid chromatography (HPLC) and mass spectrometry have been used to detect emerging contaminants [3,4,9,10,11]. Despite their effectiveness, these have significant drawbacks. They are expensive due to the high cost of equipment and reagents and require highly trained personnel. Additionally, these methods are labor-intensive and slow, limiting their use in situations requiring real-time or high-frequency monitoring.

Given these challenges, the search for more accessible and efficient alternatives is a priority in the field of contaminant detection. Surface-enhanced Raman spectroscopy (SERS) has emerged as a highly promising technique due to its selectivity, sensitivity, and non-destructive capacity, crucial in various applications, from environmental monitoring to medical diagnosis [12,13,14,15,16,17]. In this technique, an enhanced Raman signal is produced by the interaction of light with metallic nanostructures (e.g., Au, Ag, and Cu), generating the excitation of surface plasmons [14,15,18]. These excitations, known as surface plasmon resonances, increase the electromagnetic field on the surface of isolated nanoparticles or in interparticle spaces (“hot spots”) [14,15,19]. This phenomenon allows for an increase in the Raman signal of various analytes located in the vicinity of these hot spots by several orders of magnitude [14,15,19,20].

In this context, different types of SERS materials have been reported in the literature, with colloidal gold nanoparticles being particularly notable for their important and reliable SERS signal [21,22,23,24]. These nanoparticles with different shapes are synthesized by a reduction in metal salts using methods such as those proposed by Turkevich and Brust [25,26,27]. However, these reactions require highly controlled laboratory conditions and generate toxic residues, which are costly to mitigate. Additionally, many applications require arrays of gold nanoparticles on a solid substrate [28,29]. Although various strategies to immobilize nanoparticles have been implemented, such as deposition techniques and chemical methods [30,31,32], achieving ordered and precise arrays remains a major challenge. Moreover, it is very difficult to cover large areas with these strategies [33,34].

To overcome these challenges, several authors have employed top-down methodologies to obtain gold nanostructures on various substrates. Typically, these substrates, known as SERS substrates, are produced via advanced nanofabrication techniques, such as electron beam lithography, focused ion beam, and UV photolithography [15,19,35]. These methods offer high reproducibility and sensitivity, enabling precise control over the size, shape, and gap distance between metallic nanostructures [15,19,35,36,37]. However, the procedures associated with these techniques are usually long, expensive, and difficult to scale.

As a result, there has been growing interest in developing less expensive and complex methods for fabricating SERS substrates. In this context, the solid-state dewetting method emerges as a promising alternative. It is a cost-effective, simple, and fast physical method with low experimental complexity that does not produce toxic residues, in contrast to the chemical methods mentioned above. This method involves the controlled heating of a thin layer of metallic material on a solid substrate to reduce the free energy of the environment–film–substrate system, transforming the film into a set of nanoparticles or droplets [38,39,40].

In this work, we demonstrate the fabrication of gold nanostructure films as SERS substrates by thermally treating gold thin films deposited on glass substrates. These SERS substrates, obtained under various synthesis conditions, were characterized for their morphological and optical properties. Subsequently, we evaluated the direct detection of Rhodamine B as a model analyte using the SERS substrates and found that they enhance the Raman signal, achieving an average intensity enhancement with respect to the main Raman peak of RhB in a range of 5 to 123 times at 6 and 1 h, respectively.

## 2. Materials and Methods

### 2.1. Materials and Cleaning the Glass Substrate

All reagents were obtained from chemical companies and used without further purification. The glass substrates underwent a two-stage cleaning process. Solid gold of 99.9% purity was obtained from DIEGO JIMENEZ BARONA C.I. S.A.S, Cali, Colombia. Microscopy cover glass slides (25.4 × 76.2 mm) were obtained from GLASS LAB (Sylgard 184), being purchased from Bogotá DC, Colombia. Initially, the cover glass slides were resized to 10 × 10 mm substrates using a diamond dip cutter.

The cleaning process began with the preparation of a solution of acetone and isopropanol, both with 99.9% purity. The substrates were placed in this solution and cleaned in a Branson M1800H ultrasonic cleaner (Branson, Brookfield, CT, USA) for 15 min. Subsequently, the substrates were transferred to a solution of isopropanol and subjected to an additional 15 min ultrasonic cleaning cycle. This procedure aimed to eliminate any remaining impurities that could impact the sample quality.

### 2.2. Fabrication of SERS Substrate

An amount of 5 mg of gold was evaporated onto glass substrates using the thermal evaporation method to produce gold thin films. The process utilized a current of 70 A under a pression of ∼10^−4^ mbar. Subsequently, the prepared substrates were subjected to an in-air annealing treatment at 300 °C for 1 h, 3 h, 6 h, and 12 h to investigate the influence of annealing time on the SERS signal.

After annealing, a solution of Rhodamine B (RhB, C_28_H_31_C_l_N_2_O_3_) was prepared with RhB obtained from Merck, Rahway, NJ, USA (97% ≥ purity) in deionized water with a concentration of 5 ppm ($1.04\;\times \;10^{-5}$ M). Thus, 5 µL drops were deposited onto the nanostructured gold films. To facilitate the adsorption of RhB onto the film surface, the solution droplet was left in contact with the film for 24 h to air-dry before spectrum acquisition. The choice of RhB was made due to its widespread use in evaluating SERS activity [41,42,43,44].

### 2.3. Characterization

The morphology of the films was examined using a Tescan Clara Field Emission Scanning Electron Microscope, Tescan, Brno, Czech Republic (FE-SEM). Optical characterization was performed with a Jasco V-770 UV-visible, Jasco, Easton, MD, USA (UV-Vis) spectrometer to observe the localized surface plasmon resonance (LSPR) of the gold nanostructures on glass substrates. SERS measurements were conducted using a Jasco RMS 4500 Raman spectrometer. The samples were irradiated with a line laser at 457.10 nm (2.72 eV) with power set at 3.5 mW. All Raman spectra were recorded with an exposure time of 5 s and 30 accumulations to facilitate the visualization of the characteristic vibrational modes of RhB. To validate the uniformity of the SERS assay, we performed a 10 × 10 point map scan over each sample surface (1, 3, 6, and 12 h of heat treatment). Using the Jasco software SpectraManager 2.5, it was possible to obtain mean Raman measurements and the spectral change in the surface of the substrate along a specific X or Y direction on the map. In Figure S1, we show the average of the 100 spectra in the region with and without RhB for the 1 h sample. We can see a clear difference between the area with RhB, where the characteristic peaks of the molecule appear, and the area without RhB, where no Raman signal appears.

## 3. Results and Discussion

### 3.1. Characterization of Gold SERS Substrates

#### Effect of Thermal Treatment Time

**Figure 1:**
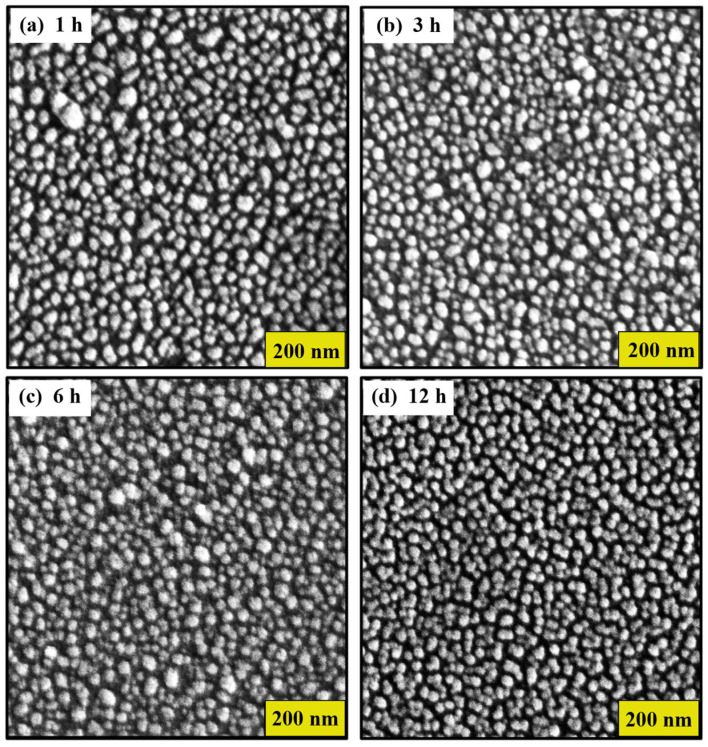
SEM images of the gold nanostructured thin films at different annealing times: (**a**) 1 h, (**b**) 3 h, (**c**) 6 h, and (**d**) 12 h.

Figure 1 shows the scanning electron microscopy (SEM) images of gold nanostructures (AuNSs) deposited on glass substrates by the thermal evaporation method. However, the glass substrates with AuNSs were annealed at a temperature of 300 °C. Here, a nanostructure with spheroidal morphology is observed, as shown in Figure 1a–c. Furthermore, as the time at the annealing temperature increases, some AuNSs tend to coalesce, as seen in Figure 1d. Despite the coalescence, it can be seen from Figure 1 that there is no major or significant difference in the size and shape of AuNSs found for samples annealed at this temperature. This suggests consistency in the annealing process in terms of nanostructure morphology.

**Figure 2:**
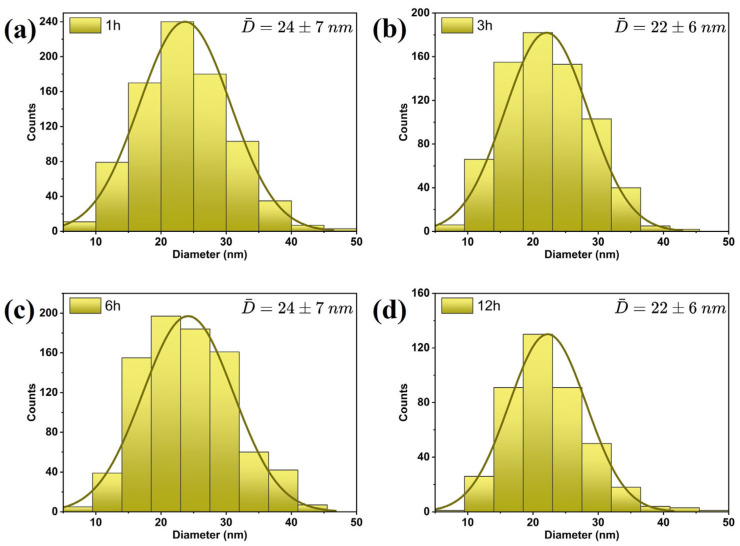
Size distribution histograms of the nanostructures: (**a**) 1 h, (**b**) 3 h, (**c**) 6 h, and (**d**) 12 h.

Furthermore, the average particle size was estimated to be between 22 ± 7 nm and 24 ± 6 nm, respectively, without considering that some nanoparticles develop irregular shapes as the annealing time increases. The estimation of the size of the nanostructures was carried out using ImageJ Software (version 1.54f). The histogram of the particle size distribution is shown in Figure 2a–d. Here, the average diameters show a variation, attributed to errors associated with the assumption of a spherical shape in the nanoparticles.

**Figure 3:**
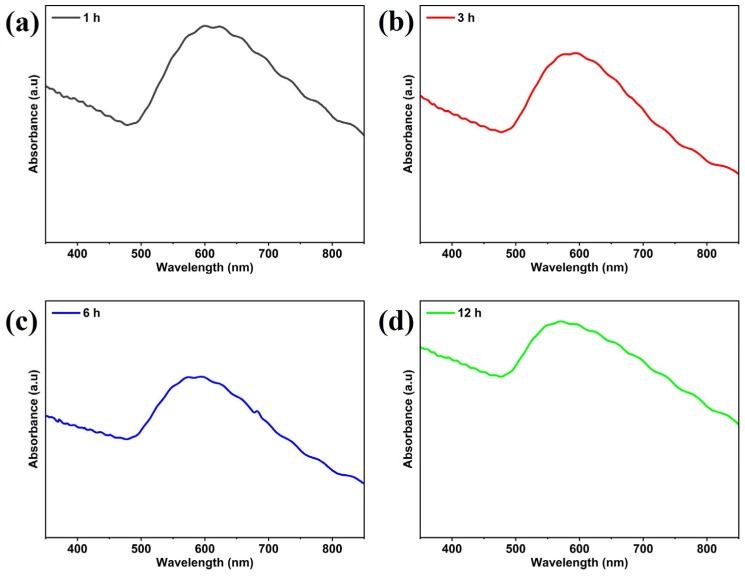
Absorption spectra of AuNSs on glass substrates annealed at 300 °C. (**a**) 1 h, (**b**) 3 h, (**c**) 6 h, and (**d**) 12 h.

On the other hand, UV–visible absorption spectroscopy was carried out to determine the localized surface plasmon resonance (LSPR) of the gold nanostructures on glass substrates. The LSPR phenomenon exhibited by AuNSs is known to be influenced by their size and shape [45]. Consequently, the absorption spectra of the film annealed for 1 h exhibited an LSPR band around 600 nm (2.07 eV), attributed to AuNSs (Figure 3a). However, for AuNSs annealed at 3 h, 6 h, and 12 h, the observed LSPR bands were located around 588 nm (2.11 eV), 590 nm (2.10 eV), and 575 nm (2.16 eV), respectively, as depicted in Figure 3c–d. Additionally, the LSPR band indicated that the supported AuNSs on the glass substrate were approximately spheroidal in shape, as observed in the SEM images. Therefore, the UV-Vis spectra for the samples annealed for 1, 3, 6, and 12 h have similar shapes. However, depending on the position of the LSPR band, a shift is observed with increased annealing time.

### 3.2. SERS Spectra of RhB Adsorbed on AuNSs

**Figure 4:**
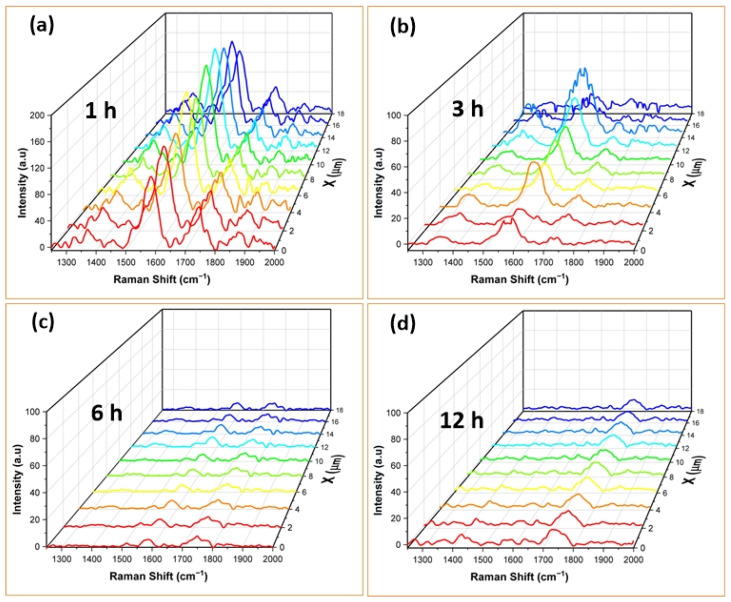
Raman spectra integrated at one point on the ‘x’ axis, across the entire ‘y’ axis spectrum sweeping a 10 × 10 μm^2^ area of RhB adsorbed on AuNSs annealed at 300 °C for (**a**) 1 h, (**b**) 3 h, (**c**) 6 h, and (**d**) 12 h.

Micro-Raman spectroscopy was utilized to characterize the surface-enhanced Raman scattering (SERS) activities of gold nanostructures fabricated on glass substrates, with Rhodamine B employed as the model analyte. Figure 4 displays the Raman signals integrated at a point on the ‘x’ axis, across the entire spectrum of the ‘y’ axis of AuNSs annealed at 300 °C for 1 h, 3 h, 6 h, and 12 h, respectively. Additionally, no SERS signals were detected for the AuNSs without RhB. Furthermore, by examining the annealing time of each SERS substrate with the Raman signal obtained from RhB, it is inferred that 1 h or 3 h is optimal for detecting the molecule’s Raman signal in this set of samples.

It is observed that the SERS spectra exhibit similarities, although certain vibrational modes appear more intense than others. This discrepancy in intensity can be attributed to the spatial orientation of the RhB molecule relative to the substrate, which influences the Raman signal. It is also important to consider that SERS sensing could be greatly affected by the size and morphology of the substrates used. Hence, the SERS response of this molecule could be attributed solely to the properties of the substrate, without additional contributions from the molecule itself [46]. The bands observed in the SERS spectra closely match those of Rhodamine B powder (see Supplementary Information, Figure S2) and are consistent with those reported in previous Raman studies [41,43,46,47,48] (see Table 1); however, some bands exhibit a slight shift and broadening, thus altering the shape of the spectrum. This behavior could be attributed to changes in the exposed crystalline facets of the gold nanoparticles as the annealing time increases, which affects the interaction between the analyte molecules and the AuNSs [49,50,51].

Taking the above into account, the Raman signal enhancement factor was calculated using the analytical enhancement factor (AEF), with the main RhB Raman peak at 1582 cm^−1^ as the reference. The AEF is a standard method for determining the enhancement factor for such systems [21,52] and is defined as follows:

$$AEF=\frac{I_{SERS}/c_{SERS}}{I_{Raman}/c_{Raman}}$$

where, $I_{SERS}$ and $I_{Raman}$ represent the intensities of the SERS and Raman signals, respectively, while $c_{SERS}$ and $c_{Raman}$ denote the concentrations of the analyte under SERS and non-SERS conditions. Since the experimental conditions for measurements under SERS and non-SERS conditions are identical, and the RhB concentrations $c_{SERS}$ and $c_{Raman}$ are equal, the AEF calculation simply consists of taking the ratio between the intensities of measurements under SERS and non-SERS conditions. In addition, the uniformity of the AuNSs was studied by calculating the relative standard deviation (RSD) with respect to the signal of the main Raman peak of the RhB around 1582 cm^−1^.

Our results indicate the average enhancement factors of 123, 26, and 5 for 1 h, 3 h, and 6 h annealing times, respectively. For the 12 h sample, the intensity of the SERS signal was comparable to the noise level, making it impossible to estimate the presence of RhB vibrational modes and, consequently, the enhancement factors for this sample. It can be observed that during 12 h of heat treatment, a coalescence (see Supplementary Information, Figure S3) effect occurs, which can lead to a reduced number of hot spots, explaining the decrease in the Raman signal as a function of heat treatment time [28].

RSD values of 18.5, 52.9, and 19.7% were also obtained for the samples treated for 1, 3, and 6 h, respectively. It is remarkable that the samples treated for 1 and 6 h present RSD values lower than 20%, indicating low dispersion in the performance of the AuNSs (see Supplementary Information, Figure S4).

## 4. Conclusions

In this study, we illustrated the potential of gold nanostructures as substrates for fabricating surface-enhanced Raman spectroscopy (SERS) platforms aimed at detecting emerging contaminants in water. Using RhB as the model analyte, we found that a significant advantage of these AuNSs lies in their ability to enhance SERS signals, without the problems associated with conventional gold nanoparticle production methods. Our results show that the sample annealed for 1 h presented the highest AEF (123 times) and the lowest RSD (18.5%), making it the optimal SERS platform of the set of samples studied. Experimental findings evidenced that the enhancement of SERS is closely linked to the coupling of particles at the nanoscale level. Furthermore, alterations observed in the SERS spectrum upon sample drying are attributed to the dynamic behavior of RhB molecules, which is potentially linked to changes in molecular adsorption and surface distribution that manifest through distinct plasmonic resonances, as well as to the effect of coalescence promoted by the increase in heat treatment time.

## Figures and Tables

**Table 1:** Assignments of vibrational modes (ν, cm^–1^) in SERS spectra of RhB.

Observed Raman Shifts						
Other Reported RhB SERS Modes	SERS (RhB/AuNPs)				Assignment	References
	1 h	3 h	6 h	12 h		
1355, 1361, 1362	1370 w	1364 m	--	--	arom C–C str	[41,43,46,47,48]
1503, 1504, 1510, 1512	1514 sh	--	--	--	arom C–C str	[41,43,46,47,48]
1526, 1532, 1533	1522 sh	--	--	--	C–H str	[43,46,47]
1560, 1572, 1575, 1580, 1585	1583 vs	1583 vs	1584 vw	--	arom C–C str	[43,46,48]

vs—very strong, s—strong, m—middle, w—weak, vw—very weak, sh—shoulder, str—stretching, arom—aromatic ring. The Raman shifts reported for SERS conditions correspond to the average main RhB peak across the surface.

## Data Availability

The original contributions presented in the study are included in the article/Supplementary Materials, further inquiries can be directed to the corresponding author.

## Supplementary Materials

The following supporting information can be downloaded at: https://www.mdpi.com/article/10.3390/nano14181525/s1, Figure S1. Average of the 100 spectra in the region with and without RhB for the 1-h sample. Figure S2. Raman spectrum of Rhodamine B powder. Figure S3. SEM images of the gold nanostructured at 1 and 12 h. Figure S4. Relative standard deviation (RSD) and Raman signal enhancement factor (AEF) as function of the annealing time.
