# Supplementary material for: The Fabrication of Gold Nanostructures as SERS Substrates for the Detection of Contaminants in Water
Source: Nanomaterials (Basel). 2024 Sep 20;14(18):1525. doi: 10.3390/nano14181525 (PMC11434667; doi:10.3390/nano14181525)
Supplement: Supplementary file 1 [file nanomaterials-14-01525-s001.zip › nanomaterials-3174942-supplementary.pdf]

Fabrication of gold nanostructures as SERS substrates for the detection of contaminants in water

Cristhian A. Visbal[1], Wilkendry Ramos Cervantes[2,3], Lorena Marín[1,3,*], John Betancourt[1,3], Angélica Pérez[3,4], Jesús E. Diosa[3,5], Luis A. Rodriguez[3,5], Edgar Mosquera-Vargas[3,5]

1 Grupo de Películas Delgadas, Departamento de Física, Universidad del Valle, Santiago de Cali, Colombia; *cristhian.visbal@correounivalle.edu.co; marin.lorena@correounivalle.edu.co; betancourt.john@correounivalle.edu.co*
2 Institución Educativa Número Dos, Maicao, La Guajira, Colombia; *ramos.wilkendry@iedosmaicao.edu.co*
3 Centro de Excelencia en Nuevos Materiales (CENM), Universidad del Valle, Santiago de Cali, Colombia; *ramos.wilkendry@iedosmaicao.edu.co; marin.lorena@correounivalle.edu.co; betancourt.john@correounivalle.edu.co; jesus.diosa@correounivalle.edu.co, luis.a.rodriguez@correounivalle.edu.co, edgar.mosquera@correounivalle.edu.co*
4 Grupo de Óptica Cuántica, Departamento de Física, Universidad del Valle, Cali A.A. 25360, Colombia; *angelica.perez@correounivalle.edu.co*
5 Grupo de Transiciones de Fase y Materiales Funcionales, Departamento de Física, Universidad del Valle, Santiago de Cali, Colombia; *jesus.diosa@correounivalle.edu.co, luis.a.rodriguez@correounivalle.edu.co, edgar.mosquera@correounivalle.edu.co*
* Correspondence: *marin.lorena@correounivalle.edu.co*

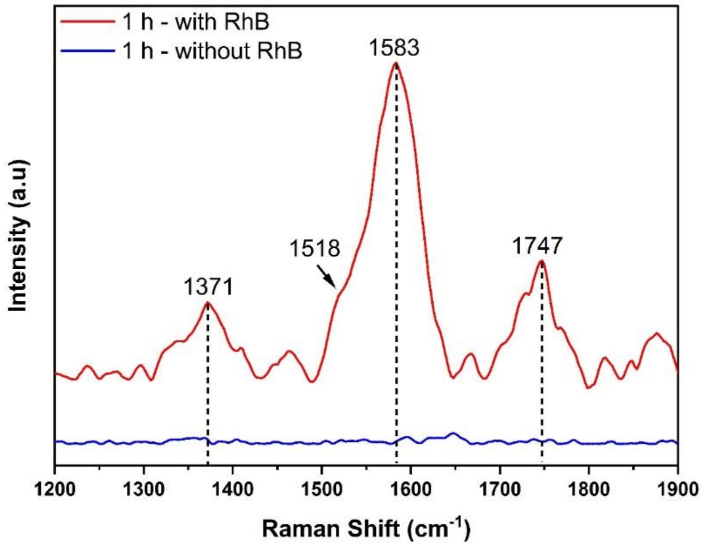

Figure S1. Average of the 100 spectra in the region with and without RhB for the 1-hour sample

In the figure S1, we show the average of the 100 spectra in the region with and without RhB for the 1-hour sample. We can see a clear difference between the area with RhB, where the characteristic peaks of the molecule appear, and the area without RhB, where no Raman signal appears.

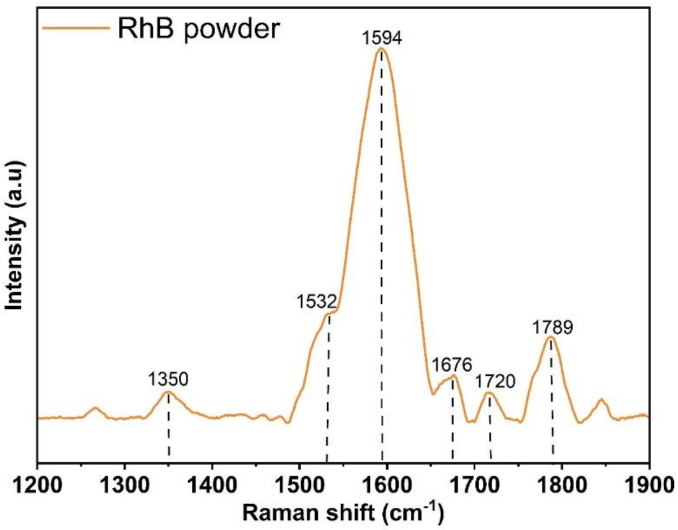

Figure S2. Raman spectrum of Rhodamine B powder.

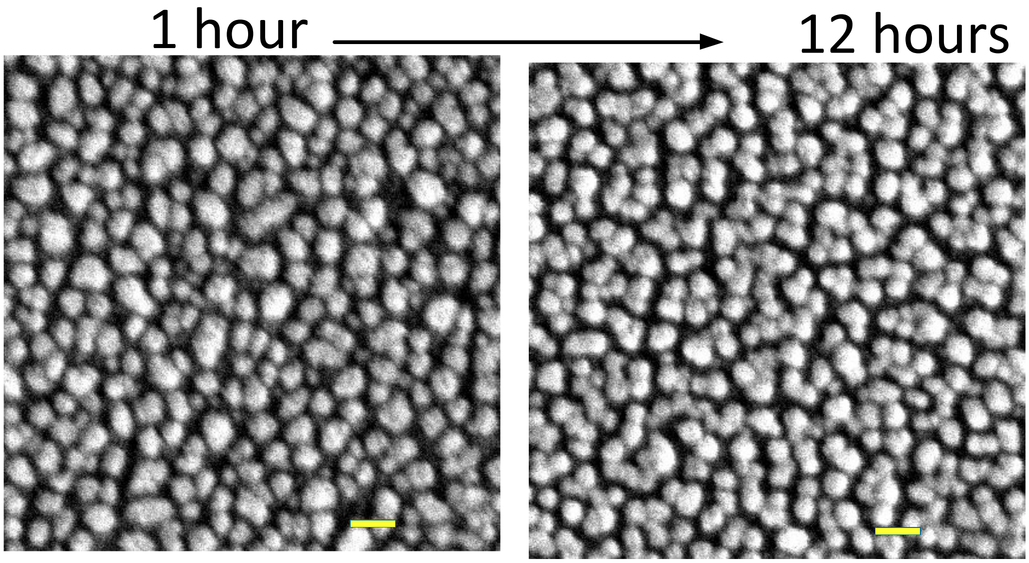

Figure S3. SEM images of the gold nanostructured at 1 and 12 hours.

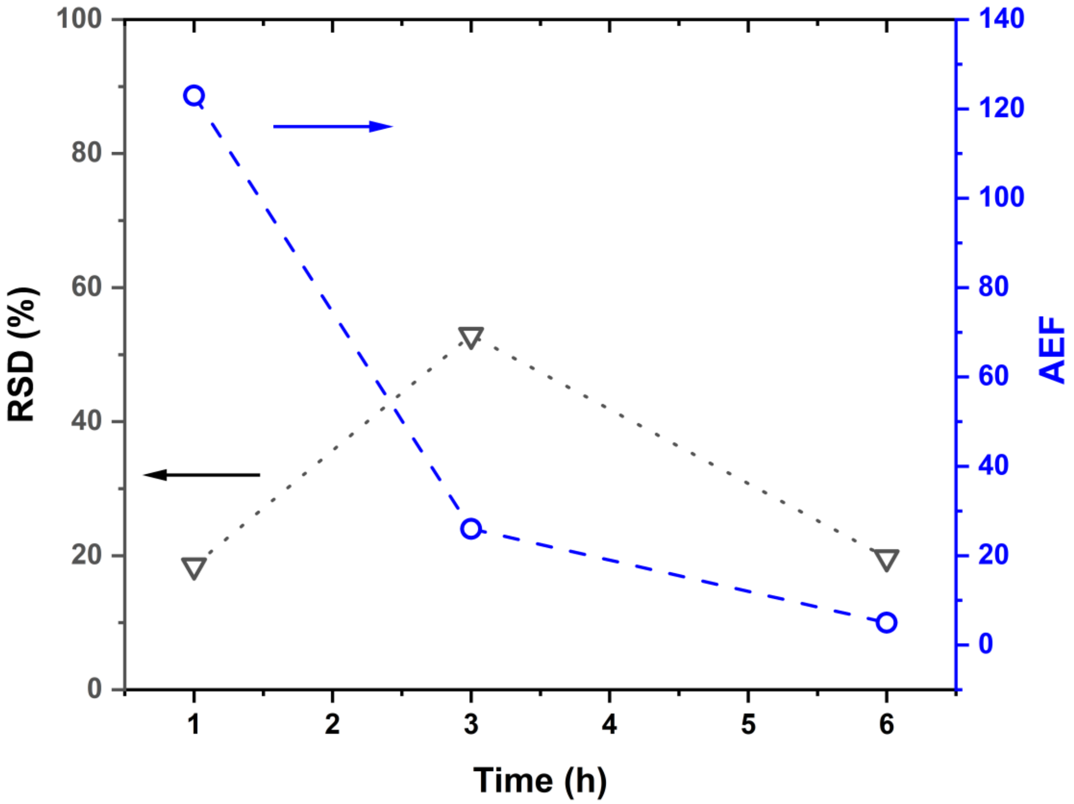

Figure S4. Relative standard deviation (RSD) and Raman signal enhancement factor (AEF) as function of the annealing time.
